# Quantification of Surface Tension Effects and Nucleation‐and‐Growth Rates during Self‐Assembly of Biological Condensates

**DOI:** 10.1002/advs.202301501

**Published:** 2023-06-06

**Authors:** Zsuzsa Sárkány, Fernando Rocha, Anna Bratek‐Skicki, Peter Tompa, Sandra Macedo‐Ribeiro, Pedro M. Martins

**Affiliations:** ^1^ IBMC − Instituto de Biologia Molecular e Celular Universidade do Porto Porto 4150–180 Portugal; ^2^ i3S − Instituto de Investigação e Inovação em Saúde Universidade do Porto Porto 4150–180 Portugal; ^3^ LEPABE − Laboratory for Process Engineering Environment Biotechnology and Energy Faculdade de Engenharia da Universidade do Porto Porto 4200‐465 Portugal; ^4^ Jerzy Haber Institute of Catalysis and Surface Chemistry Polish Academy of Sciences Niezapominajek 8 Krakow PL30239 Poland; ^5^ VIB‐VUB Center for Structural Biology Vlaams Instituut voor Biotechnology Brussels 1050 Ixelles Belgium; ^6^ Structural Biology Brussels (SBB) Bioengineering Sciences Department Vrije Universiteit Brussel (VUB) Brussels B‐1050 Belgium; ^7^ Institute of Enzymology Research Centre for Natural Sciences Budapest 1117 Hungary

**Keywords:** biological condensates, liquid‐liquid phase separation, nucleation‐and‐growth, protein aggregation, surface tension

## Abstract

Liquid‐solid and liquid‐liquid phase separation (PS) drives the formation of functional and disease‐associated biological assemblies. Principles of phase equilibrium are here employed to derive a general kinetic solution that predicts the evolution of the mass and size of biological assemblies. Thermodynamically, protein PS is determined by two measurable concentration limits: the saturation concentration and the critical solubility. Due to surface tension effects, the critical solubility can be higher than the saturation concentration for small, curved nuclei. Kinetically, PS is characterized by the primary nucleation rate constant and a combined rate constant accounting for growth and secondary nucleation. It is demonstrated that the formation of a limited number of large condensates is possible without active mechanisms of size control and in the absence of coalescence phenomena. The exact analytical solution can be used to interrogate how the elementary steps of PS are affected by candidate drugs.

## Graphical abstract

The sharp formation of large and uniformly distributed biological condensates is an intriguing observation both in the test‐tube and in cells. A solution for this apparent paradox is proposed based on the effects of surface tension during the early instants of condensate formation. A two‐parameter physical model effectively predicts the self‐assembly kinetics and size distributions of biological condensates.

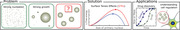

## 1 Introduction

Multiple lines of evidence indicate that liquid‐liquid phase separation (LLPS) of proteins has an important role in the spatiotemporal regulation of cell functioning and disease development.^[^ ^1^ ^]^ These findings motivate a renewed interest in the detailed description of the nucleation‐and‐growth mechanisms by which new protein phases are generated. Given the tinctorial properties of amyloid fibrils, a large number of kinetic curves have been recorded to characterize protein aggregation in vitro using thioflavin‐T and other specific dyes.^[^ ^2^ ^]^ These studies are important to understand the nucleation‐and‐growth of amyloid fibrils and other insoluble deposits associated with neurodegenerative diseases. Biophysical models such as those proposed by Crespo et al.^[^ ^3^, ^4^ ^]^ and by Finke and Watzky,^[^ ^5^, ^6^ ^]^ can describe, at least qualitatively, the hyperbolic, S‐shaped and sigmoidal progress curves usually reported for peptides and proteins such as amyloid‐*β* peptide (A*β*), *α*‐synuclein, tau, prion, transthyretin (TTR), insulin, ataxin‐3, *β*_2_‐microglobulin, huntingtin, etc. In the case of sigmoidal curves, the nucleation‐and‐growth mechanism is further validated by scaling laws of half‐life coordinates and maximal aggregation rate versus protein concentration,^[^ ^7^, ^8^ ^]^ and by measurements of particle size over time.^[^ ^9^ ^]^ Although less frequently observed, hyperbolic‐shaped progress curves characterize the amyloid aggregation of TTR,^[^ ^10^ ^]^ as well as the aggregation of A*β* at high peptide concentrations,^[^ ^11^ ^]^ or in the presence of amyloid inhibitors.^[^ ^12^ ^]^

Hyperbolic, S‐shaped and lag‐phased curves are also expected by the nucleation‐and‐growth model proposed by Zwicker et al.^[^ ^13^ ^]^ intended to describe the sigmoidal growth of centrosomes as an LLPS process controlled by an enzymatic activity of two centrioles within the centrosome matrix. Other membraneless organelles, like nuclear bodies and cytoplasmic granules undergo coacervation through the recruitment of protein and RNA molecules in supersaturated solutions, giving rise to a power‐law dependence of the average droplet size 〈*R*〉 on time *t*, 〈*R*〉∝*t^n^*. If a fixed number of growing droplets is assumed, the time exponent *n* takes on values of ≈ 1/2 in purely diffusional regimes and ≈ 1 in kinetic regimes determined by surface attachment.^[^ ^14^ ^]^ The *n* = 1/2 exponent is also predicted by the theory of diffusion‐limited precipitation, which, in addition, rationalizes the application of the Johnson–Mehl–Avrami–Kolmogorov (JMAK) equation to the early stages of LLPS progress curves.^[^ ^14^ ^]^ Both the JMAK‐ and logistic‐type equations predict that the supersaturated phase becomes fully depleted of protein for long reaction times or, equivalently, that the cellular compartment becomes completely filled with the droplet phase.^[^ ^4^, ^14^ ^]^ In order to numerically fit experimental data, these equations can be modified by the inclusion of corrected asymptotic limits for *t* → ∞,^[^ ^14^, ^15^ ^]^ whereas a power‐law relationship between 〈*R*〉(*t*) and the reaction conversion is admissible if the number of growing particles is approximately constant.^[^ ^6^ ^]^ After the nucleation‐and‐growth period has passed, the initially supersaturated phase becomes saturated and the volume fraction of the new phase increases no more. From this point onward, the average size of dense droplets in saturated solutions can continue to increase due to Ostwald ripening and/or coalescence processes in which smaller droplets convert to fewer and larger droplets driven by surface tension effects. The coalescence period that follows nucleation‐and‐growth is well characterized by a dynamic scaling exponent *n* of 1/3.^[^ ^14^ ^]^

The nucleation‐and‐growth of a few isolated particles without the continual formation of new nuclei is a challenge to the existing kinetic models with important implications for our understanding of pathogenic protein aggregation and cell organization. The time and extension of nucleation can be regulated by enzymatic activity,^[^ ^13^ ^]^ parallel oligomerization reactions,^[^ ^8^ ^]^ and ATP‐driven reactions,^[^ ^14^ ^]^ that indirectly affect the supersaturation level. New techniques and methods for studying LLPS show, however, that the formation of a reduced number of non‐coalesced condensates is possible in the absence of external sources of energy or matter.^[^ ^15^, ^16^, ^17^, ^18^, ^19^, ^20^ ^]^ To help understand these intriguing observations, we start by including surface tension effects (STEs) on established nucleation‐and‐growth models; the resulting closed‐form kinetic equations are then tested against LLPS progress curves and are used to predict the variation with time of droplet size distributions. Finally, we discuss fundamental and biological implications arising from STEs‐affected assembly and summarize the steps that should be taken to quantify STEs and nucleation‐and‐growth rates.

## 2 Thermodynamics and Kinetics of Nucleation‐and‐Growth

**Figure 1:**
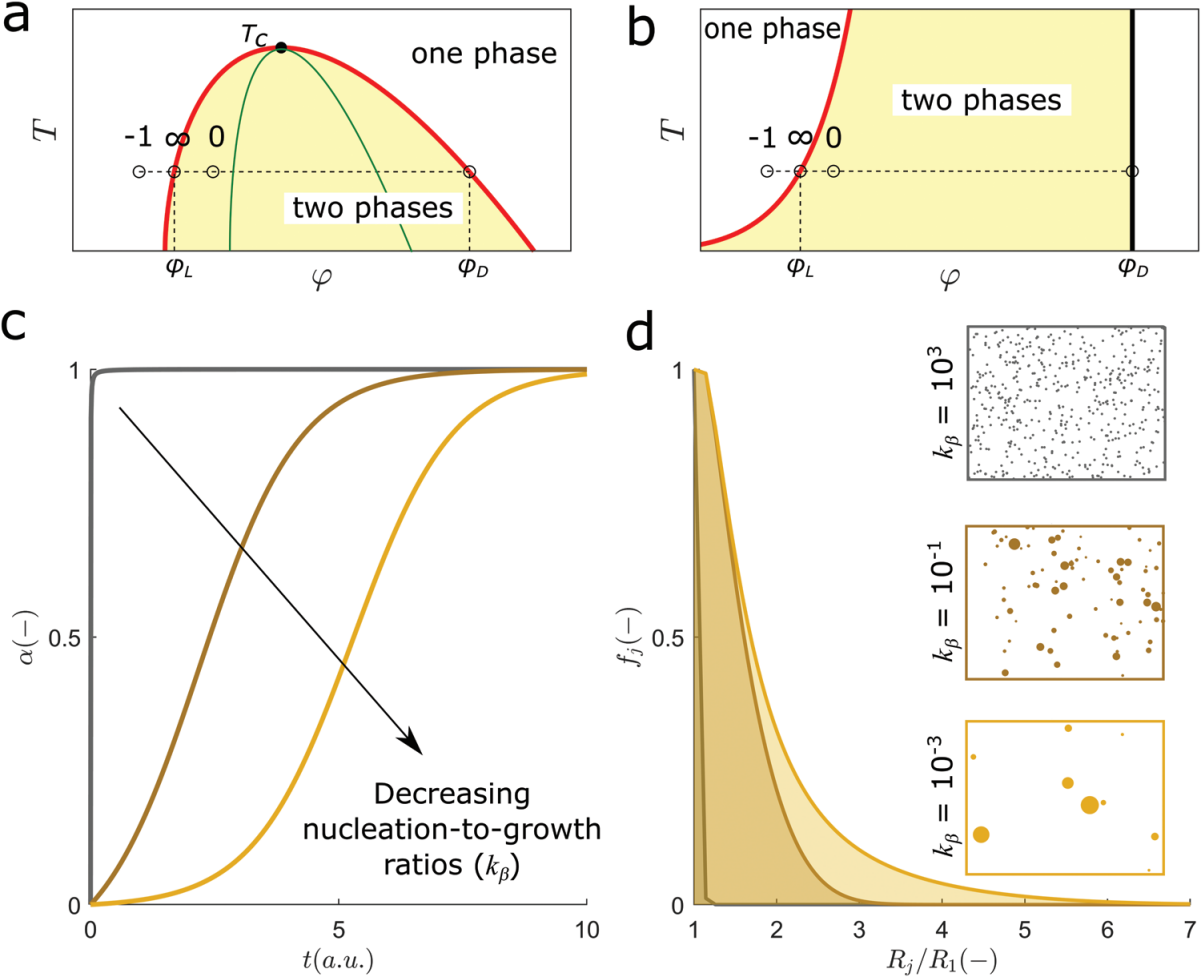
Thermodynamics and kinetics of nucleation‐and‐growth. a,b) Schematic representation of phase diagrams of binary protein‐water mixtures representing temperature (*T*) versus volume fraction of the protein (*φ*). Points along the dashed lines represent undersaturated (− 1), supersaturated (0), and saturated conditions (∞) at a fixed temperature. a) liquid‐liquid equilibrium: the volume fraction of the protein in the light (*φ*_*L*_), and in the dense (*φ*_*D*_) phase are given by the coexistence curve (red line) that shows a maximum at the critical temperature *T_c_*. Primary nucleation occurs between the coexistence curve and the spinodal line (green line). b) Liquid‐solid equilibrium: the values of *φ*_*L*_ and *φ*_*D*_ are given by solubility curve (red line) and by the solidus line (black line), respectively. c) Theoretical progress curves for nucleation‐to‐growth ratios of (from left to right) *k*_*β*_ = 10^3^ , 10^−1^ and 10^−3^ (*Supporting Information Additional Methods*). d) Normalized frequency (*f_j_*) of particles with size *R_j_* calculated for long reaction time (*t* → ∞) using the same parameters and colour code as in (c); *R*_1_ is the size of primary nuclei. Cartoon boxes: the final population of particles is schematically represented as a distribution of circles. No secondary events such as secondary nucleation, breakage or coalescence are considered in (c) and (d).

The formation of liquid protein phases follows thermodynamic and kinetic principles that are common to other phase‐transition phenomena. For example, the theoretical free energy of the mixing of binary polymer‐solvent solutions can be used to estimate the equilibrium concentrations of the protein‐depleted (or *light*) and protein‐enriched (or *dense*) phases formed upon LLPS.^[^ ^21^, ^22^ ^]^ In temperature versus composition phase diagrams, these concentrations are generally expressed in terms of the protein volume fractions *φ*_L_ and *φ*_D_, and define the so‐called *coexistence curve* or binodal (**Figure** **1**a). For attractive interactions between molecules, there is a critical temperature *T*_c_ above which phase separation does not occur, regardless of the concentration.^[^ ^23^, ^24^ ^]^ LLPS provoked by increasing temperatures can, however, occur in the case of predominantly hydrophobic interactions.^[^ ^25^ ^]^ In liquid‐solid phase diagrams, the equilibrium compositions of the light (liquid) and dense (solid) phases are given by the *solubility curve* (or liquidus) and by the solidus line, correspondingly (Figure 1b). The volume fraction of the protein in the solid phase does not change significantly with temperature and, in the case of protein crystals, can be lower than 0.5. Liquid‐liquid immiscibility can be metastable in relation to the solid phase, in which case LLPS is a precursor step of the more favorable liquid‐solid transition.^[^ ^24^, ^26^, ^27^ ^]^ According to the phase rule of thermodynamics, the number of possible microstates due to demixing increases with the number of non‐reactive components present in the mixture.^[^ ^28^ ^]^ Consequently, the complexity of the phase diagrams increases dramatically by considering the occurrence of interaction partners and metabolites, but also by including the effect of pH, ionic strength and additives,^[^ ^29^ ^]^ possible eutectic behaviors,^[^ ^30^ ^]^ the spontaneous formation of the new phase by spinodal decomposition,^[^ ^28^ ^]^ etc. In what follows, we will address the regions of protein phase diagrams where the formation of stable particles (protein crystals, aggregates or droplets) takes place by a nucleation‐and‐growth mechanism, that is, outside the region delimited by the spinodal line (Figure 1a).

Consider the change from the one‐phase to the two‐phase regime of the phase diagram by means of the isothermal increase of protein concentration from *φ*_−1_ < *φ*_L_ to *φ*_0_ > *φ*_L_. If the temperature is kept constant, the system will naturally relax toward equilibrium through the nucleation and subsequent growth of a dense phase that has constant composition (*φ*_D_) and decreases the protein volume fraction in the light phase *φ* until saturated conditions are reached ( *φ*_∞_ = *φ*_L_ ). The individual rates of the nucleation and growth steps determine how fast the change from *φ*_0_ to *φ*_∞_ is, whereas the ratio of nucleation‐to‐growth rates (*k*_*β*_) determines the shape of phase separation progress curves. In the absence of other events, increasing values of *k*_*β*_ lead to a transition from hyperbolic, to S‐shaped to lag‐phased progress curves (Figure 1c).^[^ ^3^ ^]^ In concentration units, the amount of protein separated into the new phase is *M* (*t*) = *c*_0_ − *c*(*t*), where *c*_0_ and *c*(*t*) are the initial and instantaneous protein concentrations; *M*(*t*) increases with time until reaching the final value of *M*_∞_ = *c*_0_ − *c*_∞_. In the phase diagram, *c*_0_ and *c*_∞_ correspond to volume fractions *φ*_0_ and *φ*_∞_ = *φ*_*L*_ , respectively. The instantaneous reaction conversion is *α*(*t*) = *M*(*t*)/*M*_∞_ . For large particles to be formed, primary nucleation should be much slower than the growth step (*k*_*β*_ ≪ 0.01) so that early nucleated species can continue to grow during the whole duration of lag‐phased progress curves. Because of the long period required for phase separation, large particles end up coexisting with recently formed ones that have much smaller sizes (Figure 1d). If, instead, primary nucleation is fast compared with the growth step (high *k*_*β*_), numerous small condensates will be produced until phase equilibrium is ultimately achieved (Figure 1d). In this context, the sharp formation of a limited number of large particles is an apparent paradox, whose solution is here sought using principles of phase equilibrium at curved interfaces.

## 3 Surface Tension Effects on Nucleation‐and‐Growth

We investigated how STEs might affect the early steps of phase separation. Besides driving Ostwald ripening and droplet coalescence in later stages of LLPS,^[^ ^31^, ^32^ ^]^ surface tension is also expected to influence the initial assembly of critically‐sized nuclei.^[^ ^33^, ^34^ ^]^ In fact, the condition of mechanical equilibrium between phases establishes that the difference in fluid pressure inside and outside a curved surface increases as the radius of curvature decreases. For a spherical surface of radius *r*, this positive difference is given by the Young–Laplace equation Δ*p* = 2*γ*/*r* , where *γ* is the surface tension. Consequently, the formation of primary nuclei with a radius of curvature *r* ≈ *R*_c_ is driven by a difference in chemical potential Δ*μ*_c_ that is lower than the one corresponding to a flat interface (*r* = ∞). By combining the Young–Laplace equation with the condition of chemical equilibrium between phases (Δ*μ* = 0), an equation of the Ostwald−Freundlich type is obtained,^[^ ^35^ ^]^ relating the critical protein solubility c_c_ with saturation concentration expected for flat interfaces (*c*_∞_):

(1) $$c_{c}=c_{\infty }\;\exp\left(\frac{\upsilon \gamma }{RTR_{c}}\right)$$

where υ is the molar volume of the protein and R is the universal gas constant. This equation estimates the protein solubility enhancement elicited by critically‐sized nuclei relative to larger particles characterized by *r* ≈ ∞. Taking as reference the values of *γ* = 5 × 10^−4^ J m^−2^ estimated for lysozyme crystals,^[^ ^26^ ^]^ and of υ = 0.01 m^3^ mol^−1^, a 2% increase in protein solubility is predicted for values of *R_c_* = 100 nm that are close to the initial sizes of amyloid aggregates and of liquid droplets measured by dynamic light scattering.^[^ ^8^, ^16^ ^]^ The enhancement of protein solubility will be as large as 21% if an estimate of *R_c_* = 10 nm is used in Equation (1).

The predicted magnitude of STEs is contingent on fixed parameters such as *γ* or *R_c_*, but is also greatly dependent on particle geometry premises. It is likely, for example, that flatter secondary nuclei generated at the surface of the new phase would be less affected by STEs than sphere‐like primary nuclei. On the other hand, even slight enhancements of protein solubility will significantly affect the variation in chemical potential during phase separation, which is generally approximated by the value of supersaturation.^[^ ^3^ ^]^ For small perturbations around *c*_∞_, supersaturation can be expressed in concentration units and normalized to the initial conditions (subscript 0):

(2) $$\frac{\Delta \mu \left(t\right)}{\Delta \mu_{0}}\approx \;\frac{\left(c\left(t\right)-c_{\infty }\right)/c_{\infty }}{\left(c_{0}-c_{\infty }\right)/c_{\infty }}=\frac{c\left(t\right)-c_{\infty }}{c_{0}-c_{\infty }}\;$$

When STEs affect primary nucleation, this typical definition has to be modified taking into account the critical solubility *c_c_* corresponding to the protein concentration in equilibrium with critically‐sized particles:

(3) $$\frac{\Delta \mu_{c}\left(t\right)}{\Delta \mu_{0}}\approx \left\{\begin{array}{cc} \;\frac{\left(c\left(t\right)-c_{c}\right)/c_{c}}{\left(c_{0}-c_{\infty }\right)/c_{\infty }}=\frac{c_{\infty }}{c_{c}}\;\left(\frac{\Delta \mu \left(t\right)}{\Delta \mu_{0}}\;+\;\alpha_{c}-1\right) & \mathrm{for}\;c\geq c_{c}\\ 0 & \mathrm{for}\;c<c_{c} \end{array}\right.$$

where Δ*μ*(*t*)/Δ*μ*_0_ is given by Equation (2), and the critical conversion *α*_*c*_ is:

(4) $$\alpha_{c}=\frac{c_{0}-c_{c}}{c_{0}-c_{\infty }}\;$$

As implied from the above definitions, a positive driving force for primary nucleation is kept while the protein concentration *c*(*t*) is above *c_c_* or, equivalently, while the reaction conversion *a*(*t*) is below *α*_*c*_. Thus, STEs can be quantified as the reaction extent during which no primary nucleation is observed: STEs = 1 − *α*_*c*_ .

Nucleated particles are the result of successive monomer addition events until the critical nucleus size is attained and intermolecular forces within the new phase start to prevail over the forces established with the surrounding solution.^[^ ^36^, ^37^ ^]^ Primary nucleation of macromolecules may also involve substantial structural rearrangements as in the case of amyloid fibril formation.^[^ ^38^ ^]^ Although primary nucleation is a multistep process, the stochastic assembly of molecules that leads to the formation of the primary nucleus is generally considered rate‐limiting owing to the high energy cost required for generating the new interface. If this interface has a high radius of curvature the energetic cost for nucleation is even higher due to STEs. The thermodynamics of formation of highly curved interfaces was previously investigated during the vapor‐liquid‐solid growth of nanowires by Dubrovskii and Sibirev,^[^ ^39^ ^]^ who considered that the Gibbs–Thomson effect increases the energetic barrier for nucleation. Moreover, deviations from the ideal prismatic shape of primary nuclei can increase the nucleation work and decrease the nucleation rates by orders of magnitude.^[^ ^40^ ^]^ It is assumed that once this energetic barrier is surpassed, a period of very fast growth will follow catalyzed by the exponential decrease of solubility, and will continue until the radius of curvature is fully relaxed. In such cases, the timescale for primary nucleation is still determined by the initial formation of critically‐sized nuclei, but the end‐product of the nucleation process consists of particles with size *R*_1_ larger than *R_c_*. As these particles are sufficiently flat and/or large to be insensitive to STEs, their subsequent growth will be driven by the concentration difference (*c*(*t*) − *c*_∞_), until the saturation concentration *c*_∞_ is eventually reached. Therefore, Equation (2) still defines the supersaturation during the period of growth, but we employ Equation (3) as an empirical approximation for the step of primary nucleation. Given the fast transition from critical nuclei of radius *R_c_* to stable nuclei of radius *R*_1_, the nucleation rate equations are defined assuming a constant value of critical solubility.

Following previous studies by Dubrovskii and Sibirev,^[^ ^39^ ^]^ an approximate definition of the driving force for primary nucleation is proposed to account for the enhancement of protein solubility caused by surface tension effects. This enhancement can be pronounced in the case of small, spherical nuclei but rapidly vanishes as flatter geometries are considered. If a solubility value c_c_ higher than the saturation concentration c_∞_ is considered, then primary nucleation should not occur extensively for protein concentrations below c_c_.

## 4 Exact Solutions of the General Nucleation‐and‐Growth Mechanism

To quantitatively evaluate the impact of STEs, previously established mass balance equations describing the nucleation‐and‐growth of crystals and amyloid fibrils are now modified to include the generalized definition of the driving force for primary nucleation (Equation (3) and Appendix: *General Nucleation‐and‐Growth Model*):^[^ ^4^, ^9^ ^]^

(5a) $$\begin{array}{c} \frac{1}{k_{\alpha }\Delta \mu_{0}}\;\frac{dP\left(t\right)}{dt}=\frac{M_{\infty }k_{\beta }}{N_{1}}\;{\left(\frac{\Delta \mu \left(t\right)}{\Delta \mu_{0}}\;+\;\alpha_{c}-1\right)}^{2}+\frac{k_{2}}{k_{\alpha }N_{2}}\frac{\Delta \mu \left(t\right)}{\Delta \mu_{0}}M\left(t\right) \end{array}$$

(5b) $$\frac{1}{k_{\alpha }\Delta \mu_{0}}\;\frac{dM\left(t\right)}{dt}=M_{\infty }\;k_{\beta }{\left(\frac{\Delta \mu \left(t\right)}{\Delta \mu_{0}}\;+\;\alpha_{c}-1\right)}^{2}+\frac{\Delta \mu \left(t\right)}{\Delta \mu_{0}}M\left(t\right)$$

(5c) $$N\;\left(t\right)=\frac{M\left(t\right)}{P\left(t\right)}\;$$

where *P*(*t*) is the number concentration of particles; *N*(*t*) is the mean particle size expressed in terms of number of condensed units; *N*_1_ and *N*_2_ are the number of condensed units constituting the primary‐ and secondary‐nucleation particles, respectively; and *k*_*α*_ and *k*_*β*_ are *c*_0_‐independent functions of the rate constants characterizing the microscopic steps of primary nucleation (*k*_1_), surface growth (*k*_+_) and secondary nucleation (*k*_2_):

(6a) $$k_{\alpha }=\left(k_{+}+k_{2}\right)\;$$

(6b) $$k_{\beta }=\frac{k_{1}}{c_{\infty }\left(k_{+}\;+\;k_{2}\right)}\;{\left(\frac{c_{\infty }}{c_{c}}\right)}^{2}$$

Negligible secondary nucleation rates (*k*_2_ ≈ 0) will be assumed henceforth as one of our goals is to describe nucleation‐and‐growth processes producing large condensates, whilst numerous particles with mean size close to *N*_2_ are expected in the presence of increasingly stronger secondary nucleation (Figure S4, Supporting Information). The nucleation‐and‐growth mechanism underlying the above differential equations is here called “general” because it applies to liquid‐solid phase separation, but also to LLPS processes that, in principle, are more prone to STEs provoked by the curvilinear interface of liquid droplets. Despite that, the generalizations and simplifying hypotheses adopted to derive Equations (5a and 5b) limit the applicability of this mathematical model to cases where: i) primary nucleation is described by second‐order rate equations on supersaturation, ii) growth and secondary nucleation are autocatalytic processes whose rates increase with the mass concentration of particles and are first‐order functions of supersaturation, iii) *P*(*t*) is not significantly affected by the occurrence of breakage/fragmentation events, nor by coalescence phenomena, and iv) the volume of the supersaturated solution remains approximately constant.^[^ ^4^, ^9^ ^]^

**Figure 2:**
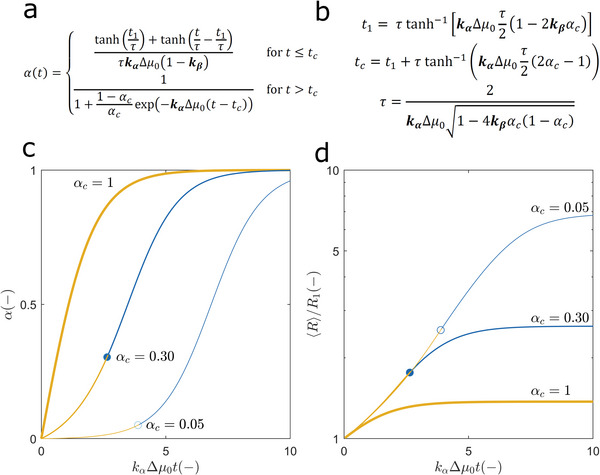
Exact solutions of Equation (5) describing the nucleation‐and‐growth of particles in the presence of surface tension effects. a) Analytical solution of Equation (5b) for the initial condition *α* (0) = 0. b) Definitions of time‐variables *t*_1_, *t_c_*, and *τ* in (a) as a function of two *c*_0_‐independent kinetic constants, *k*_*α*_ and *k*_*β*_ (in bold). c) Representation of theoretical progress curves using (from left to right) *α*_*c*_ = 1.00, 0.30, and 0.05 for a fixed nucleation‐to‐growth ratio ( *k*_*β*_ = 0.5). Lines of different color represent the nucleation‐and‐growth period (orange) and the growth‐only period (blue). Symbols indicate the location of the critical moment *t_c_*. d) Log‐linear plot of the particle growth curves obtained by numeric integration of Equation (5a) and 5c assuming the same model parameters as in (c). Symbols, line thicknesses and line colors are as in (c).

**Figure 3:**
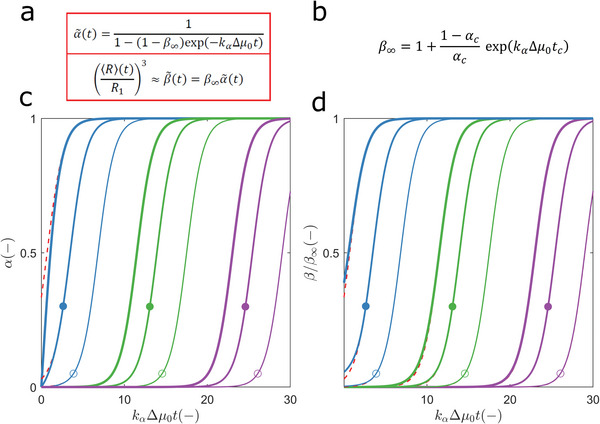
Exact and approximate solutions of Equation (5). a) The approximate $\tilde{\alpha }\left(t\right)$ and $\tilde{\beta }\left(t\right)$ expressions are derived by assuming a constant number of particles. b) From this definition of the parameter *β*_∞_, it follows that the expression for $\tilde{\alpha }\left(t\right)$ and $\tilde{\beta }\left(t\right)/\beta_{\infty }$ in (a) is equivalent to the exact *α*(*t*) solution derived for *t* > *t_c_* (Figure 2a). c) Solid lines: exact *a*(*t*) profiles computed as described in Figure 2c using *k*_*β*_ values of (different colours from left to right) 0.5, 10^−5^, and 10^−10^, and *α*_*c*_ values of (decreasing order of line thickness) 1, 0.30 and 0.05. Dashed red lines (overlapped when not seen): $\tilde{\alpha }\left(t\right)$ profiles calculated from the definitions in (a) and (b) using the same values of *k*_*β*_ and *α*_*c*_ as in the exact solution. Symbols: location of the critical coordinates *t_c_* and *α*_*c*_. d) Comparison between the exact *β* (*t*) = *N*(*t*)/*N*_1_ (solid lines) and approximate $\tilde{\beta }\left(t\right)$ (dashed red lines) solutions calculated using the same model parameters as in (c).

After replacing the definitions of Δ*μ*(*t*)/Δ*μ*_0_ = 1 − *α*(*t*) in Equation (5b), a closed‐form *α*(*t*) expression is obtained upon analytical integration (**Figure** **2**a) that is dependent on the time‐variables *t*_1_, *t_c_*, and *τ* (Figure 2b). The critical instant *t_c_* marks the transition from the nucleation‐and‐growth to the growth‐only regime and coincides with the moment the protein concentration *c*(*t*) equals *c_c_* and the reaction conversion is *α* (*t_c_*) = *α*_*c*_ ; the characteristic time constant *τ* determines the timescale for phase separation and reduces to 2(*k*_*α*_Δ*μ*_0_)^−1^ for *α*_*c*_ = 1; *t*_1_ is the instant of maximum *dα*(*t*)/*dt* slope as given by the split *α*(*t*) expression for *t* ≤ *t_c_*. Note that the products *k*_*α*_Δ*μ*_0_ *t_c_*, *k*_*α*_Δ*μ*_0_ *t*_1_ and *k*_*α*_Δ*μ*_0_ *τ* are functions of two parameters only, *k*_*α*_ and *k*_*β*_. The representation of the average particle size 〈*R*〉 as a function of *k*_*α*_Δ*μ*_0_‐normalized units of time shows that phase separation becomes slower as STEs get stronger (Figure 2c). The particle growth curves confirm that the presence of STEs induces the formation of larger particles over more prolonged timescales (Figure 2d). Similar effects are produced if different nucleation‐to‐growth ratios are considered (see **Figure** **3**c,d below). Overall, the kinetics of phase separation is determined by the initial supersaturation Δ*μ*_0_, by two kinetic parameters *k*_*α*_ and *k*_*β*_ that are independent of the initial protein concentration *c*_0_, and by the *c*_0_‐dependent value of *α*_*c*_.

The occurrence of STEs may result in the rapid nucleation and subsequent growth of a reduced number of large particles; it can also explain unconventional effects of protein concentration on the measured *α*(*t*) curves. Before illustrating these possibilities with practical examples, approximate solutions of the *N*(*t*) and 〈*R*(*t*)〉 curves are provided next toward a simple and general methodology for phase‐separation kinetic analysis.

## 5 Particle Growth Curves – Approximate Solution

Unlike the mass‐based *α*(*t*) profile, the size‐based *N*(*t*) profile resulting from the analytical solution of Equation (5) is too complex to be used in routine analyses of growth curves. An approximate expression for the size‐based profile ($\tilde{N}\left(t\right))$ is now derived assuming a constant number of growing particles *P*(*t*) ≈ *P*_∞_, which implies a final mean size of *N*_∞_ = *M*_∞_/*P*_∞_ . The dimensionless variable defining size‐based kinetics is expressed as *β* (*t*) = *N*(*t*)/*N*_1_ . The exact α(*t*) equation presented in Figure 2a for the growth‐only period can be extended to the whole nucleation‐and‐growth period (Figure 3). By combining *k*_*α*_Δ*μ*_0_, *k*_*β*_ and *α*_*c*_ in a single variable *β*_∞_, an approximate $\tilde{\alpha }\left(t\right)$ expression is obtained (Figure 3a,b) containing two model parameters (*β*_∞_ and *k*_*α*_Δ*μ*_0_) that can be numerically fitted to later stages of *α*(*t*) curves (Figure 3c). Direct replacement of this expression in Equation (5c) gives the equality $\tilde{N}\left(t\right)/N_{\infty }=\tilde{\alpha }\;\left(t\right)$, whose limit for *t* → 0 leads to the result $N_{1}/N_{\infty }=\beta_{\infty }^{-1}\;$. The expression for $\tilde{\beta }\;\left(t\right)=\tilde{N}\left(t\right)/N_{1}\;$ is appropriate for estimating *β*_∞_ and *k*_*α*_Δ*μ*_0_ from droplet size measurements (Figure 3a–d).

The differences between the exact and approximate solutions are more pronounced during the initial periods of fast nucleation processes and, as expected, are null during growth‐only periods, independently of the considered value of *k*_*β*_ (Figure 3c,d). The application of the $\tilde{\alpha }\left(t\right)$ expression to the early stages (*t* < *t_c_*) of hyperbolic progress curves is disqualified by the high errors associated with this approximation (Figure S5a, Supporting Information). On the contrary, the $\tilde{\beta }\left(t\right)$ approximation adequately describes the generality of particle growth curves (Figure S5b, Supporting Information). In the worst possible scenario, the $\tilde{\beta }\left(0\right)/\beta_{\infty }$ estimates give approximate errors of − 8% or + 8% for values of *k*_*β*_ ≈ 10^−1^ or *k*_*β*_ ≈ 10, respectively, if STEs are absent (Figure S5b, Supporting Information, inset). In all cases, initial differences between *β*(*t*) and $\tilde{\beta }\left(t\right)$ vanish as phase separation progresses (Figure 3d).

Stronger STEs or, equivalently, lower *α*_*c*_ values prolong the duration of the lag phases in an effect similar to the one elicited by decreasing values of the primary nucleation rate (compare Figures 1c and 3c). This means that sigmoidal progress curves can be the result of low *k*_*β*_ values and/or strong STEs. On the contrary, mass‐based progress curves showing hyperbolic shapes indicate fast nucleation rates and null to moderate STEs. In the next example, we demonstrate how the relative importance of STEs, *k*_*α*_ and *k*_*β*_ can be quantified from typical measurements of LLPS kinetics.

## 6 Kinetic Analysis of LLPS

**Figure 4:**
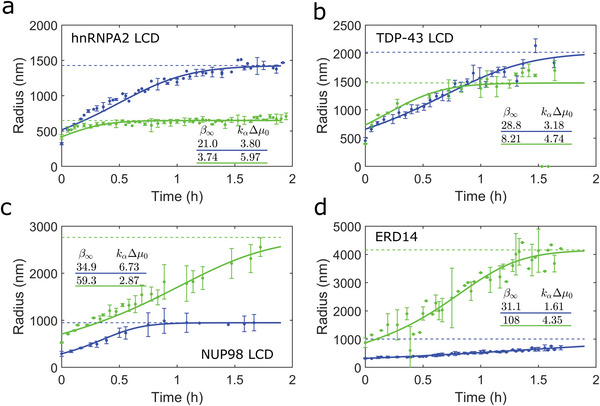
Kinetic analysis of LLPS of proteins a) 20 µm hnRNPA2 LCD, b) 80 µm TDP‐43 LCD, c) 10 µm NUP98 LCD, and d) 20 µm ERD14. Symbols: Droplet size evolution measured by Van Lindt et al.^[^ ^16^ ^]^ in the absence (blue) and presence (green) of 150 mm NaCl.^[^ ^16^ ^]^ Solid lines: fitted 〈*R*〉(*t*) curve computed as the product $R_{1}\tilde{\beta }{\left(t\right)}^{1/3}$ using the values of *β*_∞_ and *k*_*α*_Δ*μ*_0_ listed in each panel's table. Dashed lines: predicted value of the final mean size $\left\langle R\right\rangle \;\left(\infty \right)=R_{1}\;\beta_{\infty }^{1/3}$.

The new insights provided by the general nucleation‐and‐growth model are first illustrated using LLPS kinetic data obtained for the low‐complexity domain (LCD) of heterogeneous nuclear ribonucleoprotein A2 (hnRNPA2), the TAR DNA‐binding protein 43 (TDP‐43), the nuclear pore complex protein NUP98 and the Early Responsive to Dehydration 14 (ERD14) stress protein.^[^ ^16^ ^]^ These aggregation‐prone proteins can be kept stable in solution at a carefully selected extreme pH and then undergo LLPS by imposing native pH conditions through the addition of a small amount of concentrated buffer.^[^ ^16^ ^]^ Particle growth curves measured by dynamic light scattering (DLS) reveal that droplets with initial sizes > 100 nm grow at different rates in the presence and the absence of 150 mM NaCl (**Figure** **4**). As an alternative to empirical 〈*R*〉 versus *t* scaling laws, the $\tilde{\beta }\left(t\right)$ expression in Figure 3a can be used to fit the measured data considering that $\left\langle R\right\rangle \;\left(t\right)=R_{1}\;\tilde{\beta }{\left(t\right)}^{1/3}$ for invariant droplet shapes. Parameters *β*_∞_ and *k*_*α*_Δ*μ*_0_ are numerically fit to the experimental 〈*R*〉(*t*) curves using the observed initial size as an estimate of 〈*R*_1_〉. When the final size 〈*R*〉(∞) is also known, the value of *β*_∞_ can be estimated in advance from the relationship $\beta_{\infty }^{1/3}=\left\langle R\right\rangle \left(\infty \right)/R_{1}\;$. This combined parameter is affected both by *α*_*c*_ and *k*_*β*_ and represents the number of times the droplet volume is ultimately increased (Figure 3b).

The droplets produced in the different experiments are larger or smaller depending on the specific values of the model parameters originating larger or smaller values of *β*_∞_. In this respect, knowing the value of *α*_*c*_ is particularly important to ascertain the magnitude of STEs. Approximate values of *α*_*c*_ can be determined using the available estimates of the saturation concentration *c*_∞_ and critical solubility *c_c_* extracted from the 600 nm‐turbidity increase that was induced by different protein concentrations.^[^ ^16^ ^]^ Since LLPS does not spontaneously occur for *c*_0_ < *c_c_*, decreasing values of protein concentration eventually reach a point of *c*_0_ ≈ *c_c_* below which the initial turbidity signal changes no more. Accordingly, values of *c_c_* could be identified (**Table** **1**) from the protein titration results in Figure S9, Supporting Information, of the source manuscript.^[^ ^16^ ^]^ The same experimental data is used to estimate *c*_∞_ values from the x‐intercept of the maximum turbidity Δ*F*_max_ versus *c*_0_ straight line given that the final amount of the dense phase is proportional to the concentration difference Δ*c*_0_ = *c*_0_ − *c*_∞_.^[^ ^8^, ^9^ ^]^ As expected for aggregation‐prone proteins, very low values of the saturation concentration are obtained for all proteins under study (*c*_∞_ ≈ 0). Once *c*_∞_ and *c_c_* estimates are available, STEs can be quantified as STEs = 1 − *α*_*c*_ using the definition of *α*_*c*_ (Equation (4)). The presence of salts did not show a clear effect either promoting or inhibiting droplet growth. This evidence can be interpreted as the result of protein‐dependent salting‐out or salting‐in effects on the values of *c*_∞_ and *c_c_*.

**Table 1:** Model parameters estimated from the LLPS data measured by Van Lindt et al.^[^ ^16^ ^]^ and from the fitted *k*_*α*_Δ*μ*_0_ and *β*_∞_ values (shown in bold) for the cases of no added NaCl (Figure 4)

Protein	*c*_0_ [µm]	*c_c_* [µm]	STEs (−)	*t_c_* [h]	*R*_1_ [nm]	*k*_*α*_Δ*μ*_0_ [h−1]	*β*_∞_ (−)	*k*_*β*_ (−)	$\frac{k_{\alpha }\Delta \mu_{0}\tau }{2}$ (−)
hnRNPA2 LCD	20	2	0.10	1.333	517.2	3.80	21.0	0.074	1.01
TDP‐43 LCD	80	20	0.25	1.317	658.5	3.18	28.8	0.090	1.04
NUP98 LCD	10	3.8	0.38	0.598	290.2	6.73	34.9	0.086	1.04
ERD14	20	10	0.50	1.283	319.3	1.61	31.1	0.908	3.29

Besides *α*_*c*_, the nucleation‐to‐growth ratio *k*_*β*_ is a key parameter to understand why larger or smaller particles are generated. Next, we show how *k*_*β*_ can be estimated. The analysis of the $\tilde{\beta }\left(t\right)$ solution in Figure 3d and Figure S5, Supporting Information, shows that $\tilde{\beta }\;\left(t_{c}\right)=\alpha_{c}\;$ is a good approximation for all combinations of *α*_*c*_ and *k*_*β*_. Thus, our next step in the characterization of model parameters is the identification of the instant *t_c_* at which the equivalence $\left\langle R\right\rangle \left(t_{c}\right)/R_{1}=\alpha_{c}^{1/3}\;$ is verified (Figure 4). Then, the definitions of time constants *τ* and *t*_1_ in Figure 2a can be expressed as a function of parameters *k*_*β*_, *α*_*c*_ and *k*_*α*_Δ*μ*_0_, and replaced in the definition of *t_c_* to obtain the *k*_*β*_ estimate (Table 1). With the *k*_*β*_ results in hand, values of *τ* and *t*_1_ can be obtained using their mathematical definitions in Figure 2b.

Of all proteins and conditions considered in Table 1, hnRNPA2 LCD is the one less affected by STEs because the adopted initial concentration is much higher than *c_c_*. In the absence of NaCl, the mean size increase 〈*R*〉(∞)/*R*_1_ experimentally measured for this protein was higher than for ERD14, the protein more strongly affected by STEs − compare Figure 4a–d. This observation is explained by lower nucleation‐to‐growth ratios (lower *k*_*β*_ values) exhibited by the first protein. However, if LLPS had been followed over a longer period, a 31‐fold increase in droplet volume would be expected for ERD14, a value that is larger than the value of *β*_∞_ obtained for hnRNPA2 LCD (Table 1). The case of ERD14 illustrates the possibility of fast nucleating droplets ( *k*_*β*_ = 0.91) that slowly grow into micrometer‐sized particles due to the existence of a growth‐only period after *t* = *t_c_* . In contrast to what happens for the other proteins, the *τ* values listed in Table 1 for ERD14 are much larger than 2(*k*_*α*_Δ*μ*_0_)^−1^ as a consequence of more pronounced STEs and of faster nucleation rates. The reason why this protein takes more time to complete LLPS is explained by a slow growth step and by the increased importance of this step in the presence of STEs. As illustrated below in the Discussion section, strong STEs (low *α*_*c*_) and fast nucleation rates (high *k*_*β*_) uniquely combine to allow for functional phase separation via sharply regulated responses to minute concentration changes. Interestingly, the combination of stronger STEs (lower *α*_*c*_) and higher *k*_*β*_ is observed for ERD14 (Table 1), which is an intrinsically disordered chaperone with the function of helping plants to survive under dehydration stress,^[^ ^41^, ^42^ ^]^ whereas hnRNPA2, TDP‐43, and NUP98 occur in bimolecular condensates associated with disease.^[^ ^16^, ^43^ ^]^

## 7 Predictive Power

**Figure 5:**
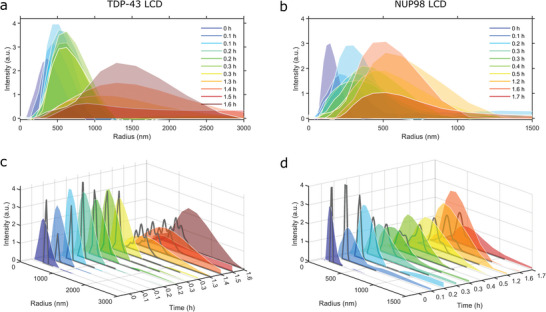
Time variation of the droplet size distributions. a,b) DLS intensity distributions obtained for a) TDP‐43 LCD and b) NUP98 LCD. c,d) Gray lines: light scattering intensities predicted by the general nucleation‐and‐growth model for c) TDP‐43 LCD and d) NUP98 LCD using the values of *α*_*c*_, *k*_*α*_Δ*μ*_0_ and *k*_*β*_ listed in Table 1. Further numerical details in *Supporting Information Additional Methods*. Different colored curves: same experimental results as in (a) and (b).

We now investigate whether the two‐parameter physical model can quantitatively predict the evolution of size distributions during the phase separation of TDP‐43 LCD and NUP98 LCD. For these proteins, the measured DLS autocorrelation functions (Figure S6, Supporting Information) yield reliable size distributions in all stages of LLPS (**Figure** **5**a,b). To compare the experimental data with the model predictions, light scattering intensities are simulated as a function of time and droplet size using the zeroth *P*(*t*), first *M*(*t*) and second *Q*(*t*) moments of the size distribution, from which the mean size *N*(*t*) (first cumulant) and the variance *σ* ^2^(*t*) (second cumulant) are determined (Appendix: *Size Distributions*). This strategy is an alternative to the recursive integration of the master equation (Equation (S2), Supporting Information) over the possible values of *j* = *N*_1_ , *N*_1_ + 1, …, *N*_∞_, a method that becomes impractical for the droplet sizes in question.

The computed curves follow a similar trend to the one observed for the mean size, shape and scattering intensity of the DLS distributions (Figure 5c,d). This fact is all the more remarkable when we consider that no numerical adjustments were required besides the choice of a fixed proportionality factor normalizing the scattering intensities during each experiment and that automatic Mie scattering corrections are applied to the simulated size distributions. Moreover, the droplet sizes are markedly distinct in the cases of TDP‐43 LCD and NUP98 LCD and spread over distinct range values as LLPS proceeds. Assuming that primary nucleation generates particles with an invariant size *R*_1_ and not with a distribution of sizes is a computational simplification that explains the narrower distributions predicted by the model as compared with the measured data (Figure 5c,d). The combination of results in Figures 4 and 5 illustrates that the general nucleation‐and‐growth model has the potential to characterize, and even predict intricate aspects of phase separation using the *α*_*c*_ measurable and two kinetic parameters.

## 8 Discussion

The sub‐critical region of the phase diagram opens surprising avenues for deciphering the origins of intriguing biological processes, from pathogenic protein aggregation to the spatiotemporal control of condensate formation in the cell. Proteins can be stable and functional under supersaturated conditions without the immanent risk of undergoing primary nucleation and forming potentially toxic aggregates. In this light, the association of certain protein misfolding disorders with ageing would not result from the natural supersaturated state of key macromolecules,^[^ ^44^ ^]^ but would rather reflect the increasing chances of the critical concentration limit *c_c_* to be crossed as the individual grows old. Such transgressions are likely to occur as the result of, for example, incidental cell stress, chemical modifications of the protein and altered proteostasis. If the nuclei resist cellular quality control mechanisms, a single species might be enough to elicit a cascade of events comprising the secondary nucleation of new assemblies and the cell‐to‐cell spreading of the disease in a prion‐like fashion.

Another possibility opened by STEs is the formation of large and uniformly distributed particles without recourse to coalescence or external players such as enzymes and chaperones. This possibility was hard to conceive from a purely biophysical point of view: either primary nucleation is fast and steady‐state conditions are rapidly achieved through the formation of numerous small particles, or primary nucleation is slow relative to the growth step and non‐uniform size distributions are obtained with very large particles coexisting with very small ones (Figure 1c,d, Movie S1, Supporting Information). The occurrence of large and uniformly distributed condensates in the cell has been enigmatic and is often ascribed to some unknown or not fully outlined regulatory process(es). However, the observation of the same pattern in test‐tube experiments demonstrates that STEs are essential for the understanding of biological phase separations. Narrow size distributions are expected in the presence of strong STEs because the occurrence of primary nucleation becomes limited to the initial moments of phase separation (Figure S3c,d and Movie S2, Supporting Information). Splitting nucleation‐and‐growth into two consecutive steps facilitates the production of large condensates with precisely controlled size distributions.

By deliberately simplifying our analysis to phase separation of binary mixtures, we demonstrate that size heterogeneity is not the inexorable result of nucleation‐and‐growth processes which, on the contrary, can be spontaneously regulated according to basic physicochemical principles of phase equilibrium. This is illustrated by the bell‐shaped size distributions that are only produced in the presence of STEs (Figure S7, Supporting Information). We consider that the quantitative tools here presented are also useful to understand how multicomponent LLPS can be accurately controlled in the cell through the modulation of interfacial properties. Being interfacially active, crucial proteins, such as G3BP1 in stress granules, MEG‐3 in P granules, centrosomin and DSpd‐2 in the centrosome, and TPX‐2 in the mitotic spindle, can induce a generalized solubility drop in the lighter phase that provokes the recruitment of all molecule whose concentration is above the critical limit. Surface tension modulation, therefore, emerges as a general organizing principle regulating phase separations in health and disease.

We envisage a practical application of our biophysical model in drug discovery, namely on the quantification of the effects of STEs‐modulators and kinetic inhibitors on the progress curves of biological self‐assembly. In a first step, the importance of STEs should be evaluated by ascertaining whether the differences between *c*_∞_ and *c_c_* are significant. As exemplified using the data measured by Van Lindt et al.,^[^ ^16^ ^]^ both quantities can be estimated from the plots of the total amount of dense phase versus protein concentration. Thermodynamic modulators are expected to change the values of *α*_*c*_ calculated using Equation (4). Kinetic modulators are identified from their effect on the reaction progress curves, which can be fitted to the exact *a*(*t*) solution (Figure 2a) or the approximate 〈*R*〉(*t*) solution (Figure 3a) depending if mass‐based or size‐based data are available. Deviations from the theoretical 〈*R*〉(*t*) curve (Figure 3a) are expected if significant secondary nucleation occurs. In that case, the exact numerical solution of Equation (5) or the approximate 〈*R*〉(*t*) solution assuming no STEs present can be adopted in the alternative.^[^ ^9^ ^]^ In principle, the *a*(*t*) solution (Figure 2a) is widely applicable independently of the magnitudes of STEs and secondary nucleation rates. Size distribution analysis may eventually reveal a continuum transition from the monomeric state, to mesoscale clusters, to the formation of a new liquid phase;^[^ ^45^ ^]^ when this happens, sharp nucleation‐and‐growth models do not apply and should be replaced by oligomerization equilibrium analysis.

Quantifying the effect of kinetic inhibitors on the rates of growth, primary nucleation, and secondary nucleation is well‐established in protein misfolding research,^[^ ^7^, ^46^, ^47^ ^]^ but it is almost absent from biological LLPS research where chemical kinetic analysis is generally based on semi‐empirical scaling laws and exponents. In addition to the *a*(*t*) and 〈*R*〉(*t*) solutions here derived, we also proposed novel methodologies to quantify STEs from the measurements of the saturation concentration *c*_∞_ and critical solubility *c_c_* (see analysis to Figure 4). We showed, moreover, that the discrete size distributions of biological condensates can be inferred at the quantitative level using kinetic parameters fitted to *a*(*t*) or 〈*R*〉(*t*) progress curves (Figure 5). This opens new possibilities for gauging the effect of phase separation modulators from measured values of equilibrium concentration and size‐distribution parameters.

## 9 Conclusion

Biological phase separation is associated with multiple aspects of cell physiology and may be conducive to neurodegenerative processes. How, when and where this phenomenon occurs remains difficult to predict even under test‐tube experimental conditions. Here we propose a physical description of protein phase separation that explains intriguing observations such as the spontaneous formation of a few condensates with large and uniform sizes. We show that the caveats in the current nucleation‐and‐growth theory can be solved by addressing the role of surface tension during the formation of primary nuclei. A “margin of safety” is introduced so that the birth of the new phase becomes thermodynamically unfavored in metastable regions of the phase diagram. The difference between the saturation limit (*c*_∞_) and the critical solubility (*c_c_*) can be interpreted in the light of the Young‐Laplace equation for curved interface equilibria. These principles apply to liquid‐solid and LLPS alike and are included in the master equation describing nucleation‐and‐growth phenomena (Equation (5) and Equation (S2), Supporting Information), whose closed‐form solution is here derived (Figure 2a). In most situations, the exact solution simplifies to an exponential‐type function describing how the average particle size changes over time (Figure 3a). The model predictions are validated using kinetic data measured for different proteins during the phase separation of different‐sized biological condensates. We found that the final size, as well as the moment of formation of biological condensates become highly regulated in the presence of strong STEs and that the evolution of droplet size distributions can be predicted based on the *α*_*c*_ measurable and two kinetic parameters (Figure 5c,d). The free parameters of the model (*k*_*α*_ and *k*_*β*_) are integrated into the variables *k*_*α*_Δ*μ*_0_ and *β*_∞_ when approximate model equations are used to fit the kinetic data. The generalized nucleation‐and‐growth model can be used for chemical kinetics analysis of isolated systems, but it could also be extended over complex, multicomponent systems for interrogating mechanisms of cell regulation and neurotoxicity involving phase separation.

## 10 Experimental Section

### Model Validation

The general nucleation‐and‐growth model and its validity were tested using kinetic data of LLPS (Figure 4) and amyloid aggregation (Figure S1, Supporting Information), by rationalizing the formation of phase‐separated condensates with large and uniform sizes under conditions where active biological processes were not present (Figure S3d and Movies S1 and S2, Supporting Information), and by comparing the predicted time evolution of droplet sizes with the measured one (Figure 5c,d). The raw data for graphs presented in Figure 4 are available in the Supplementary Data files of reference.^[^ ^16^ ^]^ The raw data presented in Figure S1, Supporting Information, were digitized by us from the original reference.^[^ ^10^ ^]^ Details of the adopted numerical methods in *Supporting Information Additional Methods*.

### DLS Measurements

Measurements of DLS autocorrelation functions were carried out on a DynaPro NanoStar (Wyatt) instrument during LLPS of 80 µm TDP‐43 LCD and 10 µm NUP98 LCD induced by pH jumps from 3.5 to 7.5 (TDP‐43 LCD) and 3.0 to 7.5 (NUP98 LCD). A disposable cuvette (WYATT technology) was filled with 100 µL of protein solution at pH and concentration values at which LLPS occurred. The sides of the cuvette were filled with water and a cap was put on top. The samples were illuminated by a 120 mW air‐launched laser of 658 nm wavelength intensity and the scattered light was recorded at a scattering angle of 90° at 25 °C, for a period of 6 h, collecting 10 acquisitions (8 s each). Each measurement was repeated at least three times. Hydrodynamic radii of the particles in solution were estimated from the diffusion coefficient delivered from the analysis of measured autocorrelation functions as described in Figure S6a,b, Supporting Information.

## Conflict of Interest

The authors declare no conflict of interest.

## Supporting information

Supporting Information

Supplemental Movie 1

Supplemental Movie 2

## Data Availability

The data that support the findings of this study are available from the corresponding author upon reasonable request.
