# Supplementary material for: Quantification of Surface Tension Effects and Nucleation‐and‐Growth Rates during Self‐Assembly of Biological Condensates
Source: Adv Sci (Weinh). 2023 Jun 6;10(23):2301501. doi: 10.1002/advs.202301501 (PMC10427409; doi:10.1002/advs.202301501)
Supplement: Supplementary file 1 — Supporting Information [file ADVS-10-2301501-s003.pdf]

ADVANCED SCIENCE
Open Access

Supporting Information

for *Adv. Sci.*, DOI 10.1002/advs.202301501

Quantification of Surface Tension Effects and Nucleation-and-Growth Rates during Self-Assembly of Biological Condensates

*Zsuzsa Sárkány, Fernando Rocha, Anna Bratek-Skicki, Peter Tompa, Sandra Macedo-Ribeiro and Pedro M. Martins**

Supporting Information (SI)

Quantification of surface tension effects and nucleation-and-growth rates during self-assembly of biological condensates

Zsuzsa Sárkány, Fernando Rocha, Anna Bratek-Skicki, Peter Tompa, Sandra Macedo-Ribeiro and Pedro M. Martins

- Appendix
    - General Nucleation-and-Growth Model
    - Size Distributions
- Additional Methods
    - Numerical Methods
- Additional Discussion
    - Kinetic Analysis of Amyloid Aggregation
    - STEs on phase diagrams and particle size distributions
- Additional Figures
    - Figure S4. The impact of secondary nucleation on the final particle size in the absence of STEs.
    - Figure S5. Maximum error of the $\tilde{\alpha}(t)$ and $\tilde{\beta}(t)$ approximations (Fig. 3a) relatively to the exact solution of Eq. 5.
    - Figure S6. DLS autocorrelation functions during LLPS of (a) TDP-43 LCD and (b) NUP98 LCD.
    - Figure S7. Using the gamma distribution to describe the exact solution of the master model equation (Eq. S2).
- SI References.

Appendix

General Nucleation-and-Growth Model: In previous master equations describing nucleation-and-growth of amyloid fibrils, the concentration of filaments composed of $j \geq N_2$ monomers (C_j) is determined by the rate constants k_1, k_2, k_+ and k_- characterizing the processes of primary and secondary nucleation, autocatalytic surface-growth, and fragmentation, respectively:[1,2]

$$\begin{aligned}\frac{\mathrm{d}C_j(t)}{\mathrm{d}t} &= \frac{1}{j}\left[k_1\Delta\mu^2(t)\delta_{j,N_1} + k_2\Delta\mu(t)M(t)\delta_{j,N_2}\right] + \\ &+ k_+\Delta\mu(t)(j-1)C_{j-1}(t) - k_+\Delta\mu(t)jC_j(t) + \\ &+ \frac{1}{j}\left[-k_-C_j(t)(j-N_2)\mathcal{H}(j-2N_1-1) + \sum_{i=j+1}^{\infty} k_-C_i(t)\mathcal{H}(j-2N_1-1)\right]\end{aligned} \qquad \text{(S1)}$$

where the Kronecker delta function sets the sizes of the primary and secondary nuclei and the Heaviside function establishes a minimum size of $2N_1 + 1$ molecules above which fragmentation starts to occur.[2] All of the rate constants k_1, k_2, k_+ describe multistep processes comprising bulk diffusion and a surface attachment step, at least.[1,3] For simplicity, the effects of particle shape on the nucleation rates are considered to affect all particles equally, while the autocatalytic dependence of the growth term on j is considered shape-independent.[1–4] Autocatalytic surface-growth was originally proposed by Finke and Watzky (F-W) to explain sigmoidal-shaped kinetic curves (describing transition-metal nanocluster formation) and implies that the elementary growth constant has to be corrected by a scaling factor given by the ratio between surface atoms increase and total atoms increase.[5] Later on, the F-W mechanism was used to describe protein aggregation either as a direct adaptation ([3] and references therein) or by considering that surface integration is preceded by the formation of critically-sized islands of adsorbed molecules.[1] In the Crespo et al. model, the linear dependence of growth rate on the mass of the growing phase results from the direct proportion between the attachment probability and the total influx of monomers into the adsorbed islands' periphery where the actives sites for growth are located.[1,6] Both the F-W and the Crespo et al. models consider that the autocatalytic surface-growth step is not diffusion limited. We further assume that STEs mainly affect the driving force for primary nucleation and thus only the k_1 term has to be expressed in relation to the critical supersaturation level ($\Delta\mu_c$):

$$\frac{\mathrm{d}C_j(t)}{\mathrm{d}t} = \frac{1}{j}\left[k_1\Delta\mu_c^2(t)\delta_{j,N_1} + k_2\Delta\mu(t)M(t)\delta_{j,N_2}\right] + \\ +k_+\Delta\mu(t)(j-1)C_{j-1}(t) - k_+\Delta\mu(t)jC_j(t) + \\ +\frac{1}{j}\left[-k_-C_j(t)(j-N_2)\mathcal{H}(j-2N_1-1) + \sum_{i=j+1}^{\infty} k_-C_i(t)\mathcal{H}(j-2N_1-1)\right] \tag{S2}$$

In the presence of STEs, the end-product of primary nucleation consists of particles with radius R_1 (and size N_1) larger than the value of critical radius predicted by the Classical Nucleation Theory. The variation in the assembly number and mass concentrations is given by the zeroth and first moments of the $C_j(t)$ distribution:

$$P(t) = \sum_j C_j(t) \tag{S3a}$$

$$M(t) = \sum_j jC_j(t) \tag{S3b}$$

and are used to obtain Eqs. 5a and 5b after differentiation, replacement of Eq. S2 and algebraic manipulation of the summations; to obtain Eq. 5a, negligible fragmentation rates are assumed, whereas in Eq. 5b the terms dependent on k_- ultimately cancel each other. Furthermore, from the definitions of $\alpha(t) = M(t)/M_\infty$, $M_\infty = c_0 - c_\infty$ and $\Delta\mu_c(t)/\Delta\mu_0$ (Eq. 3), constants k_α and k_β can be defined as a function of the elementary rate constants as follows:

$$k_\alpha = (k_+ + k_2) \tag{S4a}$$

$$k_\beta = \frac{k_1}{c_\infty(k_+ + k_2)}\left(\frac{c_\infty}{c_c}\right)^2 \tag{S4b}$$

In the absence of STEs, $c_c = c_\infty$ and $\Delta\mu_c = \Delta\mu$, which simplifies Eqs. 5a and 5b to a system of ODEs of known analytical solution:[1,2]

$$P(t) = \frac{M_\infty\alpha(t)}{N_1}\frac{k_b}{1-k_b}\left[\frac{\ln\big(1-\alpha(t)\big) + k_\alpha\Delta\mu_0 t}{\alpha(t)(1-k_b)}\left(1-\frac{k_2}{k_\alpha}\frac{N_1}{N_2}\right) - \left(1-\frac{k_2}{k_\alpha k_b}\frac{N_1}{N_2}\right)\right] \tag{S5a}$$

$$\alpha(t) = 1 - \frac{1}{k_b[\exp(k_\alpha\Delta\mu_0 t) - 1] + 1} \tag{S5b}$$

where $k_b = k_n/(k_+ + k_2)$, with $k_n = k_1/c_\infty$.[1,2]

In the presence of STEs, Eq. 5b can be solved isolatedly to obtain the following analytical solution:

$$\alpha(t) = \begin{cases} \dfrac{\tanh\left(\frac{t_1}{\tau}\right) + \tanh\left(\frac{t}{\tau} - \frac{t_1}{\tau}\right)}{\tau \boldsymbol{k_\alpha} \Delta\mu_0 \left(1 - \boldsymbol{k_\beta}\right)} & \text{for } t \leq t_c \\ \dfrac{1}{1 + \frac{1-\alpha_c}{\alpha_c} \exp\left(-\boldsymbol{k_\alpha} \Delta\mu_0 (t - t_c)\right)} & \text{for } t > t_c \end{cases} \tag{S6}$$

where

$$t_1 = \tau \tanh^{-1}\left[\boldsymbol{k_\alpha} \Delta\mu_0 \frac{\tau}{2}\left(1 - 2\boldsymbol{k_\beta}\alpha_c\right)\right] \tag{S7a}$$

$$t_c = t_1 + \tau \tanh^{-1}\left(\boldsymbol{k_\alpha} \Delta\mu_0 \frac{\tau}{2}(2\alpha_c - 1)\right) \tag{S7b}$$

$$\tau = \frac{2}{\boldsymbol{k_\alpha} \Delta\mu_0 \sqrt{1 - 4\boldsymbol{k_\beta}\alpha_c(1 - \alpha_c)}} \tag{S7c}$$

Therefore, Eq. S5b is the limit case of Eq. S6 when $\alpha_c = 1$ (no STEs present).

Size Distributions. The particle size distributions (PSDs) predicted by the general nucleation-and-growth model are obtained by recursive integration of Eq. S2 over the possible values of j. This method cannot be always adopted since heavy computational requirements are required when larger particles are considered: in the exemplary case of a 10 kDa globular protein, a small spherical droplet of $\sim$100 nm radius would contain $j > 3 \times 10^5$ monomeric units. As an alternative, the mean size $N(t)$ and variance $\sigma^2(t)$ of the $C_j(t)$ distribution can be determined as a function of the principal moments using the definitions of the first and second cumulants:

$$N(t) = \frac{M(t)}{P(t)} \tag{S8a}$$

$$\sigma^2(t) = \frac{Q(t)}{P(t)} - \left[\frac{M(t)}{P(t)}\right]^2 \tag{S8b}$$

The definition of the second moment,

$$Q(t) = \sum_j j^2 C_j(t) \tag{S9}$$

is differentiated and used together with Eq. S2 and the $P(t)$, $M(t)$ sumations (Eq. S3) to obtain:

$$\frac{1}{k_\alpha \Delta\mu_0} \frac{dQ(t)}{dt} = N_1 M_\infty k_\beta \left(\frac{\Delta\mu(t)}{\Delta\mu_0} + \alpha_c - 1\right)^2 + \frac{k_2}{k_\alpha} \frac{\Delta\mu(t)}{\Delta\mu_0} N_2 M(t) + \frac{k_+}{k_\alpha} \frac{\Delta\mu(t)}{\Delta\mu_0} (2Q(t) + M(t) - 3P(t) + 1) \tag{S10}$$

Therefore, an exact solution for the zeroth, first and second moments is possible by solving Eqs. 5a, 5b and S10 simultaneously. As described in detail in *SI Additional Methods*, the mean and variance of $C_j(t)$ are used to predict the shape of droplet size distributions measured by DLS (Figs. 6c and 6d of the main text). This is the preferred method to gain fundamental insight into all the microscopic steps involved in protein phase separation. A simpler method comprises the analysis of mass-based progress curves using the exact $\alpha(t)$ solution (Fig. 2a), which: has two free parameters (k_α and k_β); condenses the rate constants k_+ and k_2 into the parameter k_α; is independent of N_1, N_2 and k_-. A third method comprises the analysis of size-based progress curves using the approximate $\tilde{\beta}(t)$ solution (Fig. 3a), which: is valid in the absence of significant secondary nucleation and fragmentation; has two fitting parameters ($k_\alpha \Delta\mu_0$ and β_∞); condenses the influence of c_0, c_∞, c_c and of the kinetic constants in the parameter β_∞.

Additional Methods

Numerical Methods: The model equations are fitted to experimental data in Figs. 4 and S1 using the *lsqcurvefit* function of Mathworks MATLAB R2020b (Natick, MA, USA). The PSDs in Figs. 1d and S3d are generated by numerically solving Eq. S2 for long reaction times using the model parameters identified in each figure's caption assuming that $N_1 = 2$ monomers, that no condensates are initially present $C_j(0) = 0$, and a total of $M_\infty = 1000$ monomers separating into the new phase. Each particle size distribution is normalized by the maximum frequency value and represented as a function of $R_j/R_1 = \left(N_j/N_1\right)^{1/3}$. In Movies S1 and S2, the number of condensates represented in each timeframe coincides with the total number of particles calculated per unit volume; condensates composed of N_j monomers are schematically represented as circles with symbol size N_j.

To compute the $\alpha(t)$ progress curves (Figs. 1c and S3c), the system of ordinary differential equations (ODEs) comprising Eqs. 5 and Eq. S2 was numerically solved and the results expressed in $k_\alpha \Delta c_0$-normalized units of time.

The theoretical size distributions in Figs. 5c and 5d are generated from the solution of Eqs. 5a, 5b and S10 for the values of the α_c, $k_\alpha \Delta\mu_0$ and k_β parameters listed in Table 1 for TDP-43 LCD and NUP98 LCD. Negligible secondary nucleation ($k_2 \approx 0$) and the absence of initial condensates are assumed; the used R_1 values are inferred from the initial DLS distribution (450 nm for TDP-43 LCD and 160 nm for NUP98 LCD). Replacing the $P(t)$, $M(t)$ and $Q(t)$ solutions in Eq. S8 gives the variation with time of the mean size and variance of the distributions. We verified that the gamma

probability distribution adequately describes the gradual change from exponential to normal distribution predicted by the master model equation Eq. S2 (Fig. S7). The approximate $C_j(t)$ profiles are obtained from the gamma distribution function using as parameters $\theta(t) = \sigma^2(t)/N(t)$ and $k(t) = N(t)/\theta(t)$.[7] Finally, each droplet is modelled as a Mie scatterer, whose scattering intensity is calculated from the complex scattering amplitudes for two orthogonal directions of incident polarization. The MATLAB code *MatScat* was used for this purpose assuming:[8] a spherical geometry, collection of scattered light between scattering angles of 80 and 100 degrees, a complex refractive index of $1.39 + 0.01i$, a outer-medium refractive index of 1.333, a wavelength of 658 nm, and a proportionality factor of 1.11×10^{16} (TDP-43 LCD) and 1.00×10^{16} (NUP98 LCD) to normalize scattering intensity units.

Additional Discussion

Kinetic Analysis of Amyloid Aggregation: The kinetics of amyloid aggregation of monomeric transthyretin (mTTR) was fully characterized by Hurshman et al. from the increase of 400 nm-turbidity and thioflavin-T fluorescence over time.[9] Only the measurements using the amyloid-specific dye are here used to quantitatively analyze mass-based $\alpha(t)$ progress curves (Fig. S1a). In previous analyses of TTR aggregation kinetics, the experimental data was well fitted by values of $k_\beta \gg 0.01$, which, nevertheless, changed with protein concentration in a manner not expected by the Classical Nucleation Theory (CNT).[1] The applied concentrations of mTTR between 50 and 400 μg/mL are much higher than the solubility value of $c_\infty = 0.8$ μg/mL that was estimated from the monomer concentration in solution after long reaction times (1 week) independently of the departing mTTR concentration.[9] Although the terms *protein solubility* and *protein critical concentration* are often adopted interchangeably in the literature, the occurrence of STEs demands a clear distinction between c_∞ and c_c. The data measured by Hurshman et al. does not provide a direct estimate of c_c because the thioflavin-T signal becomes insensitive to protein aggregation for mTTR concentrations below 5 μg/mL.[9] Values of c_c between $c_\infty = 0.8$ μg/mL and 5 μg /mL are therefore acceptable. Model parameters k_α and k_β, and the additional unknown c_c can be numerically fitted without overparameterization problems from the analysis of the effect of protein concentration on phase separation kinetics (Fig. S1a). The obtained value of $c_c = 4.06$ μg/mL implies that the magnitude of STEs is low: $\alpha_c = 0.93$ from Eq. 4 using as c_0 the lowest concentration tested (50 μg/mL). This low magnitude is, in principle, expectable for amyloid fibrils that are generated as one-dimensional aggregates and negligible nucleation barriers.[10,11] A single pair of concentration-independent kinetic constants is enough to globally fit the measured effects of mTTR concentration on the $\alpha(t)$ time-course curves (Fig. S1a).

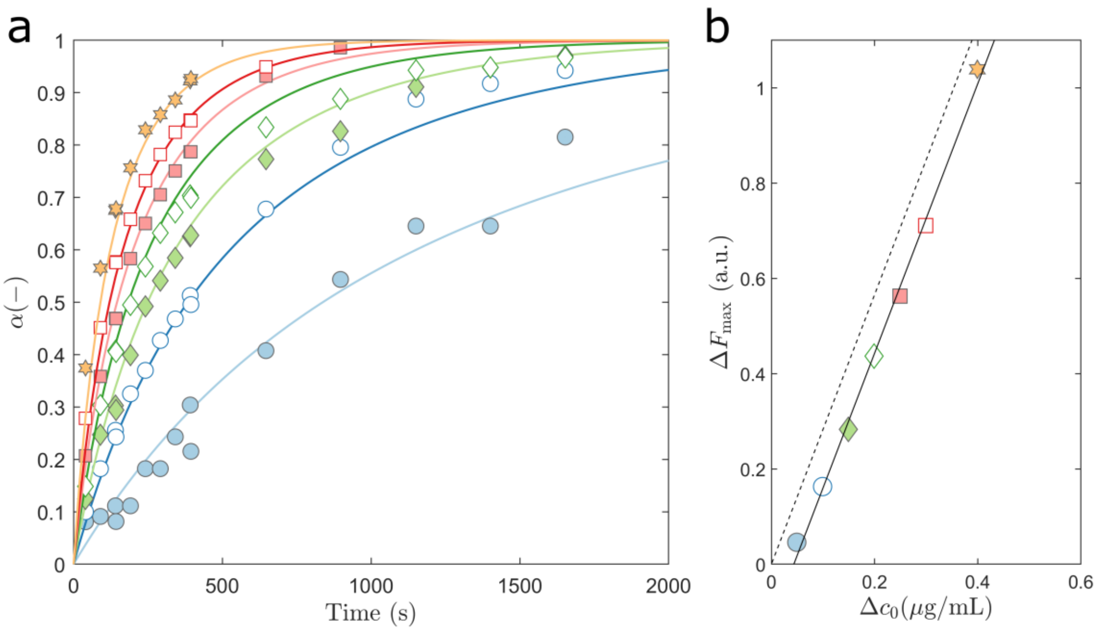

Figure S1. The general nucleation-and-growth model describes the (a) kinetics and (b) thermodynamics of mTTR aggregation measured by Hurshman et al.[9] (a) Symbols: continuous fluorescence data digitized by us into periodic data and normalized by the end-point signal for mTTR concentrations of (from top to bottom) 400, 300, 250, 200, 150, 100 and 50 μg/mL. Solid lines: Progress curves predicted by Eq. S6 for the tested mTTR concentrations using the fitted parameters $k_\alpha = 1.05 \times 10^{-2}$ s^{-1}, $k_\beta = 1.71$ and $c_c = 4.06$ μg/mL. (b) Symbols: Values of maximum fluorescence measured as a function of $\Delta c_0 = (c_0 - c_\infty)$. Solid line: the experimental results follow a linear correlation. Dashed line: example of a straight line passing in the origin.

To cross-check the validity of protein-aggregation kinetic analyses, the maximum fluorescence signal obtained at the end of the reaction ΔF_{max} should be evaluated against the concentration difference $\Delta c_0 = (c_0 - c_\infty)$. A direct proportion between these two quantities is expected in the absence of parallel aggregation pathways.[1,12] While a linear relationship between ΔF_{max} and Δc_0 is confirmed, the positive X-intercept suggests the occurrence of TTR in other forms than monomers or amyloid fibrils (Fig. S1b). Confirming this indication, significant amounts of TTR dimers were identified by Hurshman et al. using analytical gel filtration and SDS-PAGE techniques.[9] Overall, the results in Fig. S1 confirm that the effect of protein concentration on mTTR aggregation is well described by standard nucleation theories modified to include STEs.

A distinguishing feature of the general nucleation-and-growth model is the predicted effect of protein concentration on the shape of reaction progress curves. Expectedly, in the case of fast-nucleating

proteins such as mTTR, lowering the initial concentration not only slows down the rates of PS as it will also change the shape of the $\alpha(t)$ curves from hyperbolic to sigmoidal. This new possibility is admissible when the time interval during which primary nucleation occurs is significantly reduced by the occurrence of strong STEs. As we have seen for mTTR aggregation, only weak STEs are present in the range of protein concentrations studied by Hurshman et al.[9] If the formation of amyloid fibrils of mTTR could be detected accurately for concentrations lower than 50 μg/mL, magnitudes of STEs higher than 0.07 ($\alpha_c < 0.93$) could be explored, and a lag period of apparently no aggregation would become visible as c_0 gradually approached the value of $c_c = 4.1$ μg/mL (Fig. S2, dashed lines). Based on this property, it is possible to gauge the intensity of STEs by graphically representing the reaction conversion as a function of Δc_0-normalized units of time: in principle, superimposing $\alpha(\Delta c_0 t)$ curves indicate that STEs are either weak or absent (Fig. S2).

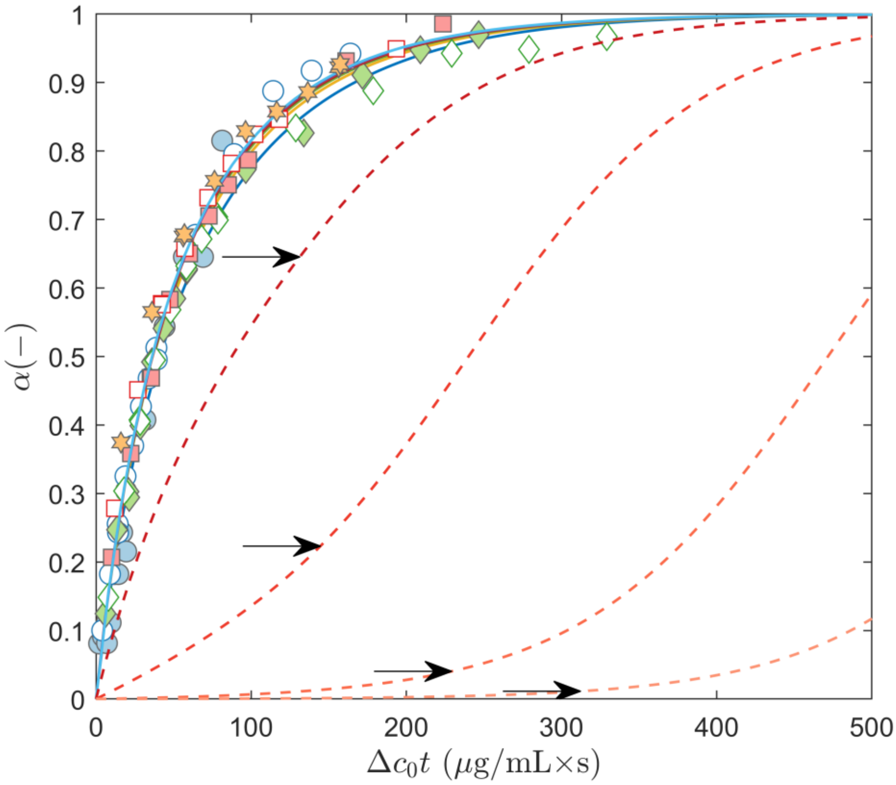

Figure S2. Identification of STEs from the representation of progress curves in Δc_0-normalized units of time. Symbols and solid lines: the results of Fig. S1a are now represented in normalized time units. Weak STEs influencing mTTR aggregation are confirmed by superimposing $\alpha(\Delta c_0 t)$ curves. Dashed lines: predicted $\alpha(\Delta c_0 t)$ curves for c_0 values of (from left to right) 10, 5, 4.2 and 4.1 μg/mL using the fitted parameters $k_\alpha = 1.05 \times 10^{-2}$ s^{-1}, $k_\beta = 1.71$ and $c_c = 4.06$ μg/mL. Arrows: critical

reaction conversions (α_c). The shape of the curves becomes concentration-dependent as protein concentrations approach c_c.

STEs on phase diagrams and particle size distributions: In terms of the CNT, the limit below which phase separation does not occur has been interpreted in relation to awaiting times arbitrarily chosen, below which nucleation is practically arrested.[13] In the presence of STEs, a thermodynamic (rather than kinetic) metastable zone is proposed where infinitely long times are required for primary nucleation to take place (in blue in Figs. S3a and S3b). In this sense, the width of the metastable zone is a parameter that can be objectively measured and is independent of the method applied to follow phase separation. Without the "margin of safety" created by STEs, protein self-assembly would be almost inevitable in the metastable environment of the cell.[14]

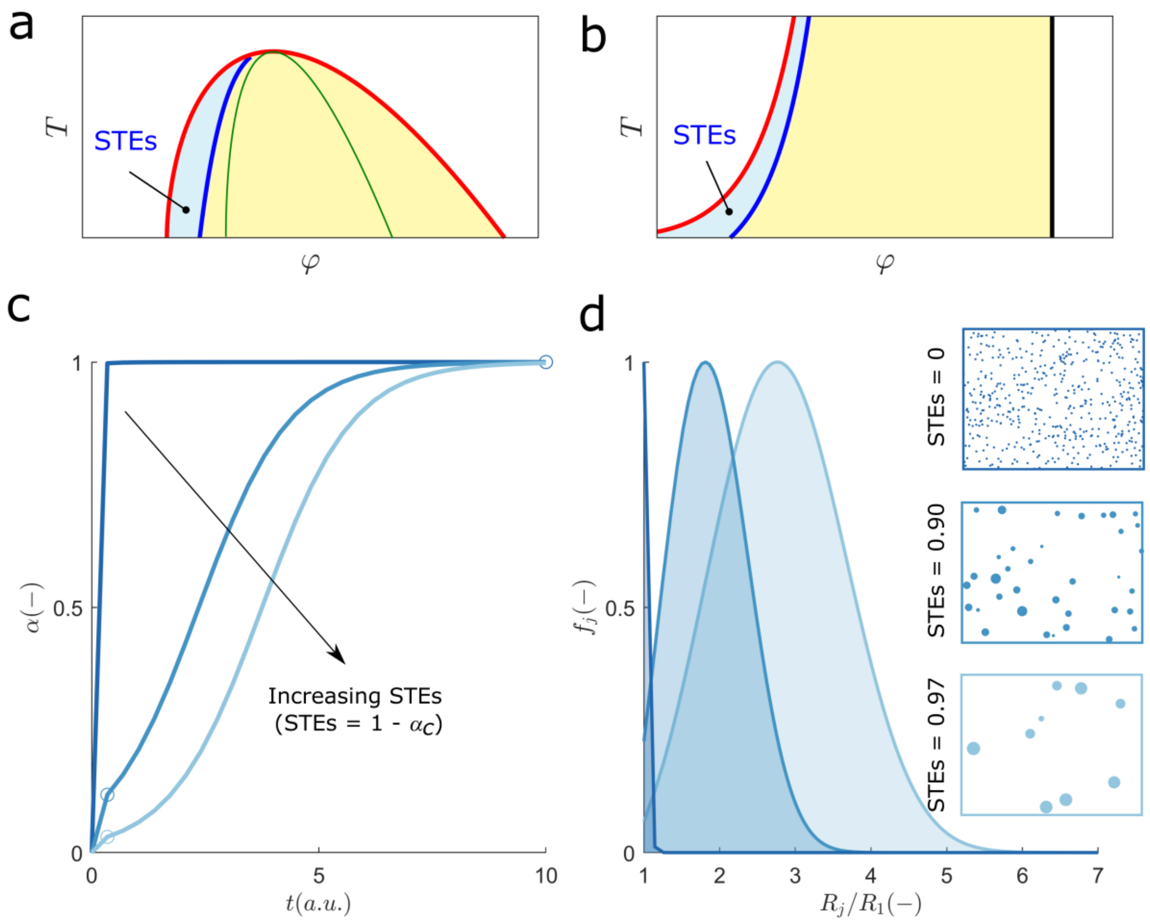

Figure S3. Thermodynamics and kinetics of nucleation-and-growth affected by STEs (refer to Fig. 1 of the main text for the case of no STEs present). (a and b) In the presence of STEs, primary nucleation becomes thermodynamically unfavored in the metastable region (blue area) of (a) liquid-liquid and (b) liquid-solid phase diagrams. (c) Theoretical progress curves calculated using $k_\beta = 10^3$ and (from left to right) STEs $= 0$, 0.90 and 0.97. Open circles: location of the critical coordinates t_c

and α_c. (d) Final f_j distribution calculated using the same model parameters and colour code as in (c). Large particle sizes uniformly distributed around the mean size can be produced in the presence of strong STEs (compare with Fig. 1d). Further numerical details in *SI Additional Methods*.

In the presence of strong STEs, a long growth-only period follows the short nucleation-and-growth period (Fig. S3c) giving rise to a small number of large particles (Fig. S3d). As we tend to the extreme of $\text{STEs} = 1$, the initial formation of a single nucleus would be sufficient to lower the supersaturation level below the critical limit and phase transition would produce only one very large condensate. Movies S1 and S2 are time-lapse simulations showing how particles self-assemble until reaching the equilibrium size distributions in Fig. 1d and S3d, respectively. From comparing the two situations, we conclude that the combination of fast nucleation with strong STEs (movie S2, $\text{STEs} = 0.97$) gives rise to a few large particles and, at the same time, leads to a sharp control of the moment new particles are formed. These features of spatiotemporal regulation of phase separation are not possible in the case of no STEs present: although large condensate bodies can still be produced under nucleation-limited conditions (Fig. 1d), new nuclei will keep arising erratically throughout the whole duration of phase separation (movie S1, $k_\beta = 5 \times 10^{-3}$).

Additional Figures

a

$$\left(\frac{N_\infty}{N_1}\right)^{-1} = \frac{k_b}{1-k_b}\left[\frac{\ln(1/k_b)}{(1-k_b)}\left(1-\frac{k_2}{k_\alpha}\frac{N_1}{N_2}\right)-\left(1-\frac{k_2}{k_\alpha k_b}\frac{N_1}{N_2}\right)\right]$$

b

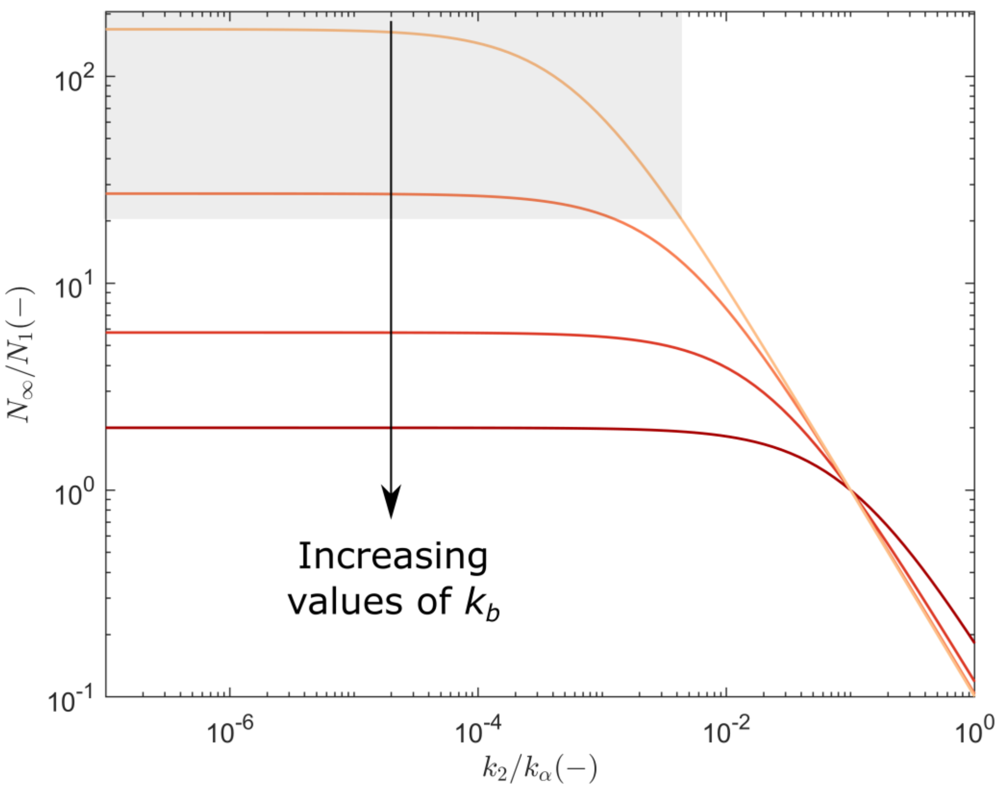

Figure S4. The impact of secondary nucleation on the final particle size in the absence of STEs. (a) The steady-state average particle size N_∞ can be calculated from Eqs. 5c and 5b extrapolated for long reaction times ($t \rightarrow \infty$). (b) The N_∞/N_1 ratio is calculated as a function of the k_α-normalized value of k_2 for values of k_b of (from top to bottom) 10^{-3}, 10^{-2}, 0.1, and 1 using $N_2/N_1 = 0.1$. Primary and secondary nucleation have to be much slower than growth for large particles to be formed (i.e., low values of both k_b and k_2/k_α are required to reach the shaded area).

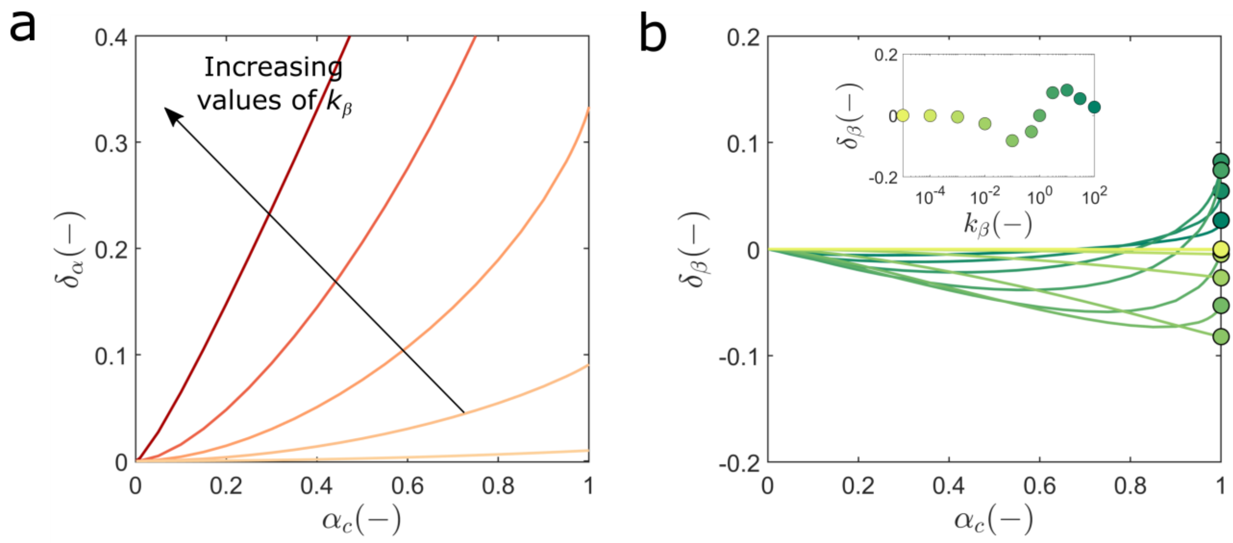

Figure S5. Maximum error of the $\tilde{\alpha}(t)$ and $\tilde{\beta}(t)$ approximations (Fig. 3a of the main text) relatively to the exact solution of Eq. 5. (a) Initial difference $\delta_\alpha = \tilde{\alpha}(0) - \alpha(0)$ for unseeded reactions ($\alpha(0) = 0$). The estimates of $\tilde{\alpha}(0) = \beta_\infty^{-1}$ are calculated from the definitions of β_∞ (Fig. 3b) and t_c (Fig. 2b) for values of k_β of (from lighter to darker shades of red) 10^{-2}, 10^{-1}, 0.5, 3 and 10^2. (b) Initial difference $\delta_\beta = \tilde{\beta}(0)/\beta_\infty - N_1/N(\infty)$, where $\tilde{\beta}(0) = 1$ (Fig. 3a) and $N_1/N(\infty)$ is numerically calculated for values of k_β from (lines from lighter to darker shades of green) 10^{-5} to 10^2. Symbols: higher values of δ_β are obtained for α_c values close to 1. Inset: the selected δ_β values are represented as a function of k_β.

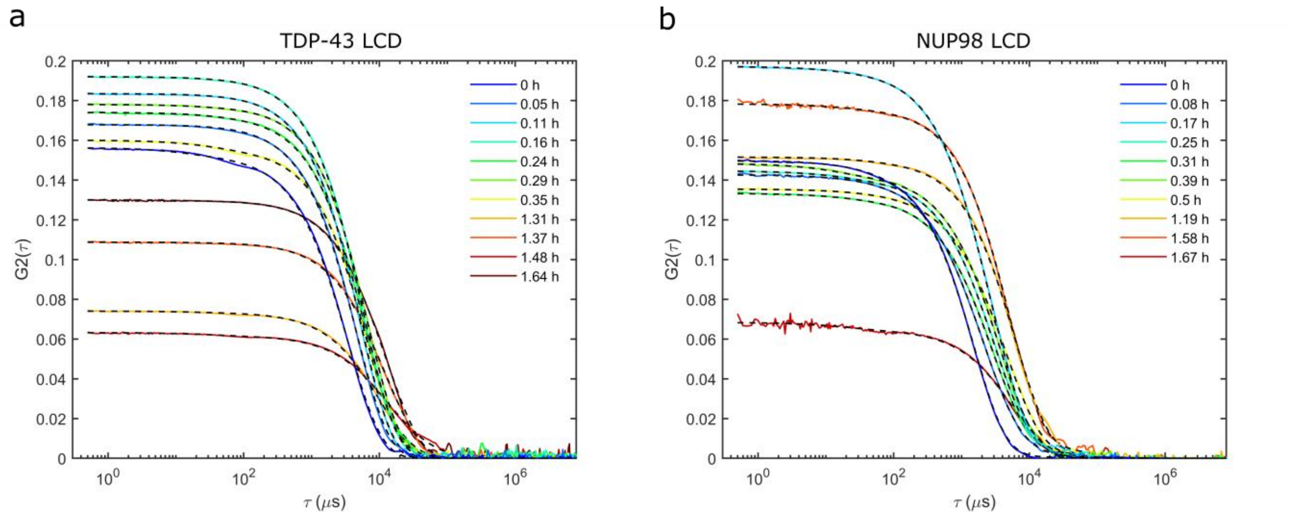

Figure S6. DLS autocorrelation functions during LLPS of (a) TDP-43 LCD and (b) NUP98 LCD. Solid lines: measured data from which the size distributions in Figs. 6a and 6b are inferred. Dashed lines: CONTIN analysis of the autocorrelation functions using the MATLAB code *rilt*.[15,16]

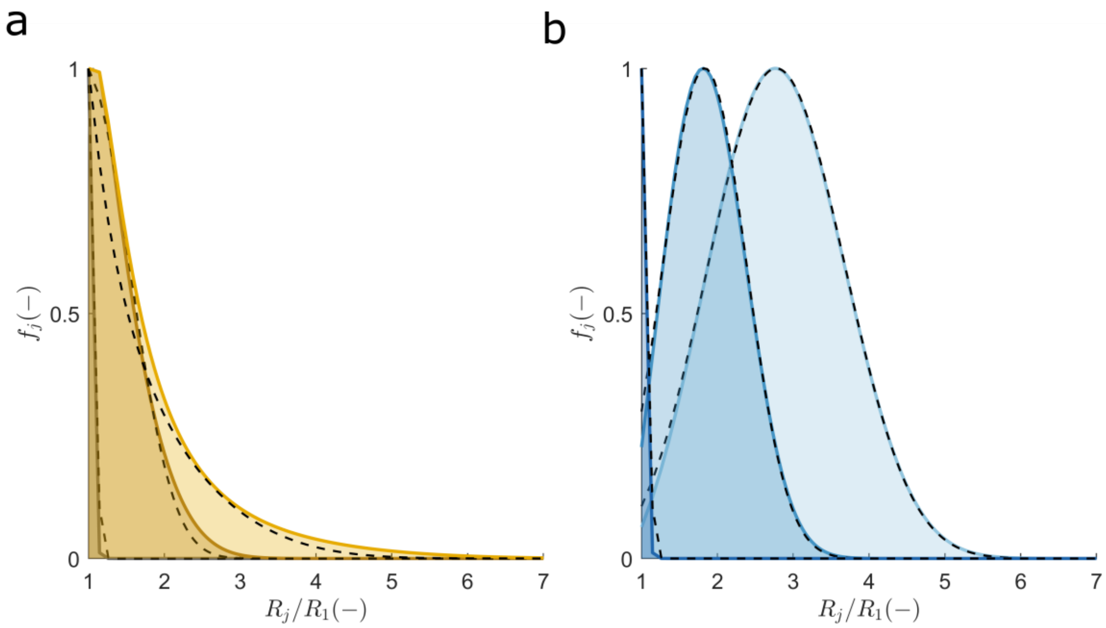

Figure S7. Using the gamma distribution to describe the exact solution of the master model equation (Eq. S2). The endpoint $C_j(t \to \infty)$ distributions obtained as in (a) Fig. 1d and (b) Fig. S3d are numerically fitted (dashed lines) by the two-parameter gamma distribution and represented in normalized frequency units. (a) In the absence of STEs, exponential size distributions are produced. (b) In the presence of STEs, bell-shaped size distributions can also be produced.

SI References

[1] R. Crespo, F. A. Rocha, A. M. Damas, P. M. Martins, *J. Biol. Chem.* **2012**, *287,* 30585.

[2] A. Silva, B. Almeida, J. S. Fraga, P. Taboada, P. M. Martins, S. Macedo-Ribeiro, *Angew. Chem. Int. Ed.* **2017**, *56*, 14042.

[3] M. A. Watzky, E. E. Finney, R. G. Finke, *J. Am. Chem. Soc.* **2008**, *130*, 11959.

[4] R. Cabriolu, D. Kashchiev, S. Auer, *J. Chem. Phys.* **2010**, *133,* 225101.

[5] M. A. Watzky, R. G. Finke, *J. Am. Chem. Soc.* **1997**, *119,* 10382.

[6] P. M. Martins, F. Rocha, *Surf. Sci.* **2007**, *601,* 3400.

[7] E. W. Weisstein, "Gamma Distribution." From MathWorld--A Wolfram Web Resource. https://mathworld.wolfram.com/GammaDistribution.html. **2022.**

[8] J. Schäfer, *MatScat* (https://www.mathworks.com/matlabcentral/fileexchange/36831-matscat), MATLAB Central File Exchange. **2022**.

[9] A. R. Hurshman, J. T. White, E. T. Powers, J. W. Kelly, *Biochemistry* **2004**, *43*, 7365.

[10] D. Kashchiev, S. Auer, *J. Chem. Phys.* **2010**, *132,* 215101.

[11] T. M. Phan, J. D. Schmit, *Biophys. J.* **2022**, *121*, 2931.

[12] A. Silva, Z. Sárkány, J. Fraga, P. Taboada, S. Macedo-Ribeiro, P. Martins, *Biomolecules* **2018**, *8*, 108.

[13] D. Kashchiev, G. M. van Rosmalen, *Cryst. Res. Technol.* **2003**, *38*, 555.

[14] G. G. Tartaglia, S. Pechmann, C. M. Dobson, M. Vendruscolo, *Trends Biochem. Sci.* **2007**, *32*, 204.

[15] I.-G. Marino, *rilt* (https://www.mathworks.com/matlabcentral/fileexchange/6523-rilt), MATLAB Central File Exchange. **2022**.

[16] S. W. Provencher, *Comput. Phys. Commun.* **1982**, *27*, 229.
